# Cost-effectiveness analysis of lenvatinib plus pembrolizumab compared with chemotherapy for patients with previously treated mismatch repair proficient advanced endometrial cancer in China

**DOI:** 10.3389/fphar.2022.944931

**Published:** 2022-09-30

**Authors:** Zhiwei Zheng, Liu Yang, Siqi Xu, Huide Zhu, Hongfu Cai

**Affiliations:** ^1^ Department of Pharmacy, Cancer Hospital of Shantou University Medical College, Shantou, China; ^2^ Department of Pharmacy, Fujian Medical University Union Hospital, Fujian Medical University, Fuzhou, China

**Keywords:** cost-effectiveness, 309-KEYNOTE-775 clinical trial, advanced endometrial cancer, lenvatinib, pembrolizumab, chemotherapy

## Abstract

**Aims:** This study aimed to evaluate the cost-effectiveness of lenvatinib plus pembrolizumab (LP) vs. chemotherapy for patients with previously treated mismatch repair proficient advanced endometrial cancer in China.

**Methods:** A lifetime of partitioned survival Markov was used to evaluate the overall lifetime, total costs, quality adjusted life years (QALYs), and incremental cost effectiveness ratio (ICER) across a 10-years time horizon in the study 309–KEYNOTE-775 clinical trial. Direct costs and utility values were gathered from available literature. The willingness to pay (WTP) was defined at $37,663.26 per QALY. Sensitivity analyses were carried out to determine the model’s uncertainty.

**Results:** According to the baseline analysis, the LP group gained 4.02 total life years and 3.13 QALYs for $93,496.69, whereas the chemotherapy group gained 2.86 total life years and 2.24 QALYs for $30,578.04. LP versus chemotherapy resulted in an incremental cost of $62,918.65, with an ICER of $70,962.09/QALY, which was higher than China’s WTP threshold ($37,663.26/QALY). The ICERs were most sensitive to the cost of pembrolizumab and the cycle of LP delivered, according to the sensitivity analysis. However, changing the range of those parameters has no influence on the model’s results.

**Conclusion:** Our present analysis suggests that LP treatment is not cost-effective for patients with previously treated mismatch repair proficient advanced endometrial cancer. However, LP treatment may be a cost-effective treatment option if the price is reduced.

## Introduction

Endometrial cancer (EC) is the most common cancer in women around the world. EC is the sixth most frequent cancer in women, accounting for 4,17,000 new cases and 97,000 deaths in 2020 (Sung et al., 2021). When patients were diagnosed with EC, 10%–15% were already at an advanced stage of the disease (Brooks et al., 2019). The prognosis of advanced EC is poor, with a 5-years survival rate of fewer than 17% among patients with distant metastases (Zhang et al., 2019). Despite advancements in EC therapy, effective therapeutic choices for people who have already had advanced endometrial cancer are limited (Braun et al., 2016). There was no standard of care for advanced or recurrent endometrial cancer following failed platinum-based chemotherapy (Abu-Rustum et al., 2021; Concin et al., 2021). Therefore, treating advanced EC has become an increasingly tough challenge, and new therapeutic options are urgently required.

In recent years, immunotherapy has emerged as an attractive treatment option and has shown good performance (Christofi et al., 2019; Musacchio et al., 2020). Among them, pembrolizumab provided an overall response rate of 57% in patients with noncolorectal high microsatellite instability or mismatch repair-deficient cancer (Marabelle et al., 2020). Another KEYNOTE-028 study showed pembrolizumab has a favorable safety profile and durable antitumor activity in a subgroup of patients with heavily pretreated advanced PD-L1-positive endometrial cancer (Ott et al., 2017). Later, the FDA authorized pembrolizumab plus lenvatinib (LP) for the treatment of patients with advanced endometrial cancer that is not microsatellite instability-high (MSI-H) or mismatch repair deficient (dMMR) and who have progressed after prior systemic therapy but are not candidates for curative surgery or radiation (Arora et al., 2020). In the phase 2 clinical Study 111-KEYNOTE-146 study, patients with advanced endometrial cancer who had previously received treatment responded favorably to lenvatinib and pembrolizumab (LP) treatment. High-grade adverse events were handled with supportive care and dosage adjustments, and there was a reasonably low incidence of withdrawal owing to adverse events. LP demonstrated significant anticancer efficacy in patients with advanced endometrial carcinoma (Makker et al., 2020).

In the phase 3 clinical trial 309-KEYNOTE-775 (Makker et al., 2022), patients with previously treated advanced endometrial cancer who received LP had significantly longer progression-free survival (PFS) and overall survival (OS) than those who received chemotherapy. However, the high expense of LP might have far-reaching economic effects. The purpose of our study was to explore the cost-effectiveness of LP compared with chemotherapy for patients with previously treated mismatch repair-proficient (pMMR) advanced endometrial cancer based on the study 309–KEYNOTE-775 trial from the perspective of the Chinese healthcare system.

## Methods

### Patients and interventions

The target population of this study is consistent with 309–Keynote-775 Trial. The clinical efficacy and safety data were based on the patients in the study 309–KEYNOTE-775 trial. Women who met the criteria for inclusion had disease progression following the administration of one prior platinum-based chemotherapy treatment and had no previous exposure to therapies that target PD-1 or vascular endothelial growth factor. Patients may have had two lines of platinum-based chemotherapy if one was used as neoadjuvant or adjuvant treatment. Regarding prior hormonal treatment use, there were no restrictions. Other prerequisites for participation were having at least one RECIST, version 1.1-measurable lesion, accessible biopsy samples for determining MMR status, and an Eastern Cooperative Oncology Group (ECOG) performance-status score of 0 or 1 (Makker et al., 2022).

Between 11 June 2018 and 3 February 2020, the 309-KEYNOTE-775 clinical trial randomized 827 patients (697 in the pMMR population and 130 in the dMMR population) to the treatment arm at 167 study centers in 21 countries. The median duration of treatment with lenvatinib plus pembrolizumab was 231 days (range, 1–817) and chemotherapy was 104.5 days (range, 1–785) (Makker et al., 2022).

The drug doses and Treatments were based on the patients in the study 309–KEYNOTE-775 trial. Patients aged 18 or older with confirmed advanced, recurrent, or metastatic endometrial cancer and mismatch repair–proficient (pMMR) disease were randomly assigned (1:1) to receive lenvatinib (20 mg, orally once daily) plus either pembrolizumab (200 mg every 3 weeks) or chemotherapy (doxorubicin 60 mg/m^2^ every 3 weeks or paclitaxel 80 mg/m^2^ weekly, with a 3 weeks on and 1 week off cycle).

As the numbers of patients receiving doxorubicin or paclitaxel was not defined in 309–KEYNOTE-775 trial, our model assumed that the patients had an equal opportunity to receive doxorubicin or paclitaxel. We will perform a sensitivity analysis of the chemotherapy opportunity to evaluate the sensitivity impact on economic outcomes.

The grade 3 or 4 adverse events were chosen from the 309–KEYNOTE-775 trial based on two criteria: 1) More than 10% of grade 3 or 4 adverse events occurred in the pabolizumab or chemotherapy groups; 2) the difference between the two groups was greater than 5%.

### Model structure

**FIGURE 1:**
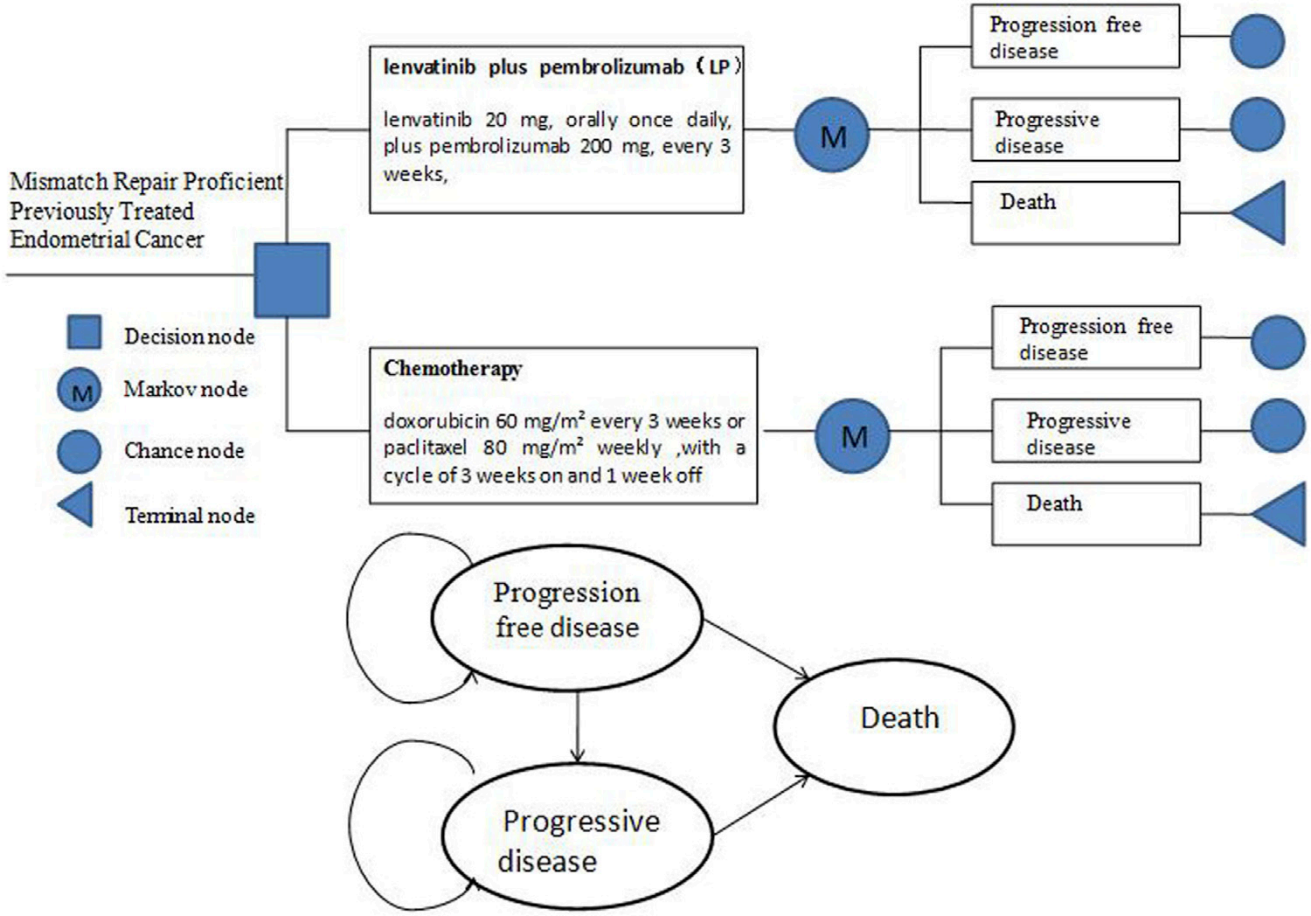
Markov Structure of three health states: progression-free disease, progressive disease and death.

A lifetime of partitioned survival Markov model was constructed using the TreeAge Pro 2015 software (Williamstown, MA, United States) to simulate the advanced endometrial cancer disease process. There were three mutually exclusive health states in the model: progression-free disease (PFD), progressive disease (PD), and death. The model’s period horizon was set at 10 years since the overall 5-years survival rate for patients with advanced EC is less than 17%. The model duration was set to 3 weeks based on the treatment in the study 309–KEYNOTE-775 trial. The median duration of treatment with lenvatinib plus pembrolizumab was 231 days (range, 1–817) and chemotherapy was 104.5 days (range, 1–785) (Makker et al., 2022) (Figure 1).

The Kaplan-Meier survival curve, generated from the study 309-KEYNOTE -775 trial using Get Data Graph Digitizer 2.25, was used to calculate the probability of transition between the three health stages. The data collected was reconstructed by R software, and the probability of survival was estimated utilizing Weibull, Log-logistic, Log-normal, Gompertz, Exponential, and Gamma distributions. Visual examination and the lowest values of the Akaike information criterion (AIC) and the Bayesian information criterion (BIC) (Hoyle and Henley, 2011) were used to identify the most suitable distribution. The log-logistic distribution function was selected to simulate the PFS and OS curves given critical scrutiny of both schemes (Supplementary Table S1; Supplementary Figures S1–S4). S(t) = 1/(1 + λt^γ^) was used to calculate the survival function of a log-logistic rate over a period (Diaby et al., 2014). The estimated scale (*λ*), shape (*γ*) and key clinical parameters were presented in Table 1.

**TABLE 1:** Model economic parameters and the range of the sensitivity analysis.

Variable		Baseline value	Range		Distribution	Source
			Minimum	Maximum		
Log-logistic survival model	PFS					
LP group		shape (*γ*) = 1.6294 scale (*λ*) = 0.04528	—	—	—	Makker et al. (2022)
Chemotherapy group		shape (*γ*) = 1.941 scale (*λ*) = 0.06522	—	—	—	Makker et al. (2022)
Log-logistic survival model	OS					
LP group		shape (*γ*) = 1.5396; scale (*λ*) = 0.01375	—	—	—	Makker et al. (2022)
chemotherapy group		shape (*γ*) = 1.8582; scale (*λ*) = 0.01024	—	—	—	Makker et al. (2022)
LP group SAEs (grade ≥ 3)incidence %						
Hypertension		37.9	—	—	—	Makker et al. (2022)
Anemia		6.2	—	—	—	Makker et al. (2022)
Neutropenia		1.7	—	—	—	Makker et al. (2022)
chemotherapy group SAEs grade ≥ 3 incidence %						
Hypertension		2.3	—	—	—	Makker et al. (2022)
Anemia		14.7	—	—	—	Makker et al. (2022)
Neutropenia		25.8	—	—	—	Makker et al. (2022)
Drug cost per mg, US $						
Lenvatinib per mg		4.255	2.7575	4.255	Gamma	Yao (2022)
Pembrolizumab per mg		25.98	12.99	25.98	Gamma	Yao (2022)
Doxorubicin per mg		0.3220	0.1958	0.4482	Gamma	Yao (2022)
Paclitaxel per mg		0.1138	0.0692	0.1584	Gamma	Yao (2022)
Costs of SAEs per cycle ($)						
Hypertension		12.9	11.6	14.2	Gamma	Wu et al. (2012)
Anemia		73.68	55.27	92.11	Gamma	Zhang et al. (2020)
Neutropenia		461.5	415.4	507.7	Gamma	Wu et al. (2012)
Other costs, US $						
Best supportive care cost per cycle		55.6	27.8	83.4	Gamma	Qiao et al. (2021)
Follow-up cost per cycle		337.50	168.75	506.25	Gamma	Qiao et al. (2021)
Health utilities						
Progression-free disease		0.817	0.797	0.836	Beta	Thurgar et al. (2021)
Progressive disease		0.779	0.699	0.859	Beta	Thurgar et al. (2021)
Hypertension		0.1	0.1	0.15	Beta	Barrington et al. (2021)
Anemia		0.074	0.037	0.11	Beta	Chou et al. (2020)
Neutropenia		0.2	0.15	0.25	Beta	Kang et al. (2021)
Body surface area, m^2^		1.64	1.288	1.96	Beta	Yue et al. (2021)
Discount rate		0.05	0	0.08	Beta	Yue et al. (2021)

LP, lenvatinib plus pembrolizumab; OS, overall survival; PFS, progression-free survival; SAEs, serious adverse events.

### Costs and utilities

The model only included direct medical expenses, such as the cost of LP and chemotherapy, treatment-related grade 3-4 serious adverse events (SAEs) management, the cost per cycle of salvage treatment, and routine follow-up. To calculate the dose of chemotherapy agents, we assumed that the average patient weighed 60 kg and was 160 cm tall, leading to a body surface area (BSA) of 1.64 m^2^. All prices were derived from local charges or previously published literature. Palliative chemotherapy and treatment options were unclear following the failure of current treatment, and the specific treatment was not demonstrated in the 309–KEYNOTE-775 study. As a result, the best support therapy was considered to be intervention after progression. Because the 309–KEYNOTE-775 study lacked data on quality of life, the utility values for the PFD and PD health states were derived from the literature.

The costs and utility were discounted at a rate of 5% according to the practice of pharmacoeconomic evaluation guidelines for universal health coverage in China (Yue et al., 2021).

All expenditures were computed in US dollars, given an RMB exchange rate of $1 to 6.45 Yuan on average for the entire year of 2021. In addition, three times the Chinese gross domestic product (GDP) in 2021 ($37,663.26) was used as the willingness to pay (WTP) threshold according to recommendations (Yue et al., 2021). All costs and utilities are presented in Table 1.

### Sensitivity analysis

To identify the most substantially impacted parameters, one-way analyses were performed on the impact of different factors on ICER when varied to a range of 25% of the base case value. The current price of pembrolizumab has changed by 50% lower. The one-way sensitivity analysis findings were displayed in the form of a tornado diagram.

The probability sensitivity analysis (PSA) was conducted using a 10,000 Monte Carlo simulation with parameters adjusted to a statistical distribution. Scatter plots and cost-effectiveness acceptability curves were used to display the PSA results.

## Results

### Base case analysis

The lenvatinib plus pembrolizumab group gained 4.02 total life years and 3.13 QALYs at a cost of $93,496.69 over a 10-years time horizon, while the chemotherapy group gained 2.86 total life years and 2.24 QALYs at a cost of $30,578.04. The incremental cost of lenvatinib plus pembrolizumab was $62,918.65 when compared to chemotherapy, with an incremental effectiveness of 0.89 QALYs and an ICER of $70,962.10/QALY (Table 2). Lenvatinib plus pembrolizumab was not a cost-effective treatment option when compared to chemotherapy alone at the Chinese cost-effectiveness WTP threshold of $37,663.26/QALY.

**TABLE 2:** The results of the cost-effectiveness analysis.

Treatment	Total cost ($)	Total life years	Total QALYs	Incremental cost ($)	Incremental QALY	ICER ($/QALY)
Lenvatinib plus Pembrolizumab	93496.69	4.02	3.13	62918.65	0.89	70962.09
Chemotherapy	30578.04	2.86	2.24	—	—	—

ICER, incremental cost–effectiveness ratio; QALY, quality-adjusted life year.

### Sensitivity analyses

**FIGURE 2:**
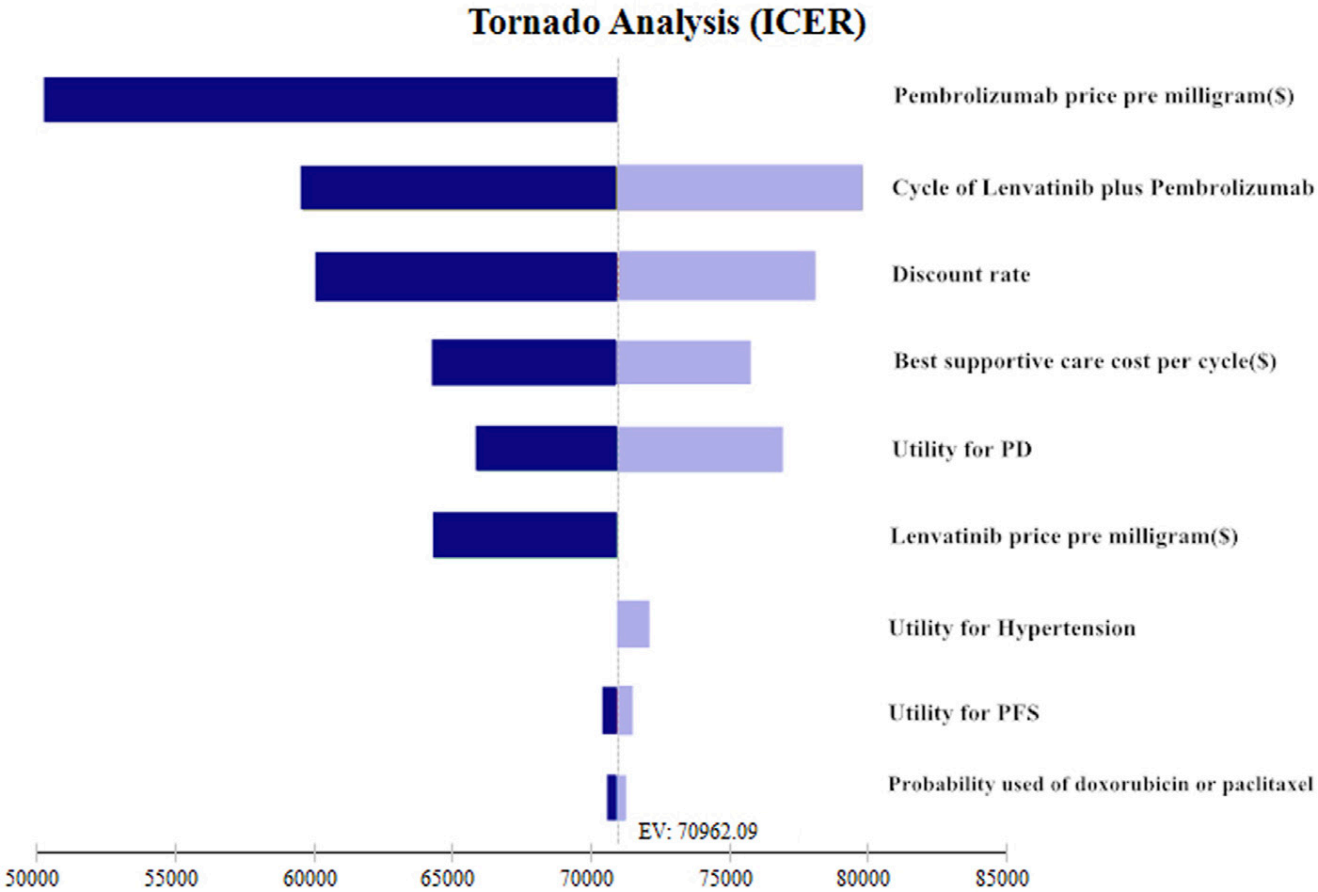
Tornado diagrams of one-way sensitivity analyses.

One-way sensitivity analysis tornado diagram was showed in Figure 2. The most influential parameters were the cost of pembrolizumab, cycle of lenvatinib plus pembrolizumab used. However, changing those two factors did not result in significant changes in the ICER to below the WTP thresholds. ($50,198.02–$70,962.09/QALY and $59,638.72–$80,063.5600/QALY, respectively.) Other factors influencing the model were the discount rate, best supportive care cost per cycle ($), utility for PD, lenvatinib price pre milligram ($), utility for hypertension, utility for PFS, probability used of doxorubicin or paclitaxel. Whereas, none of those variables could reduce the ICERs below the thresholds. All the variables did not change the results.

**FIGURE 3:**
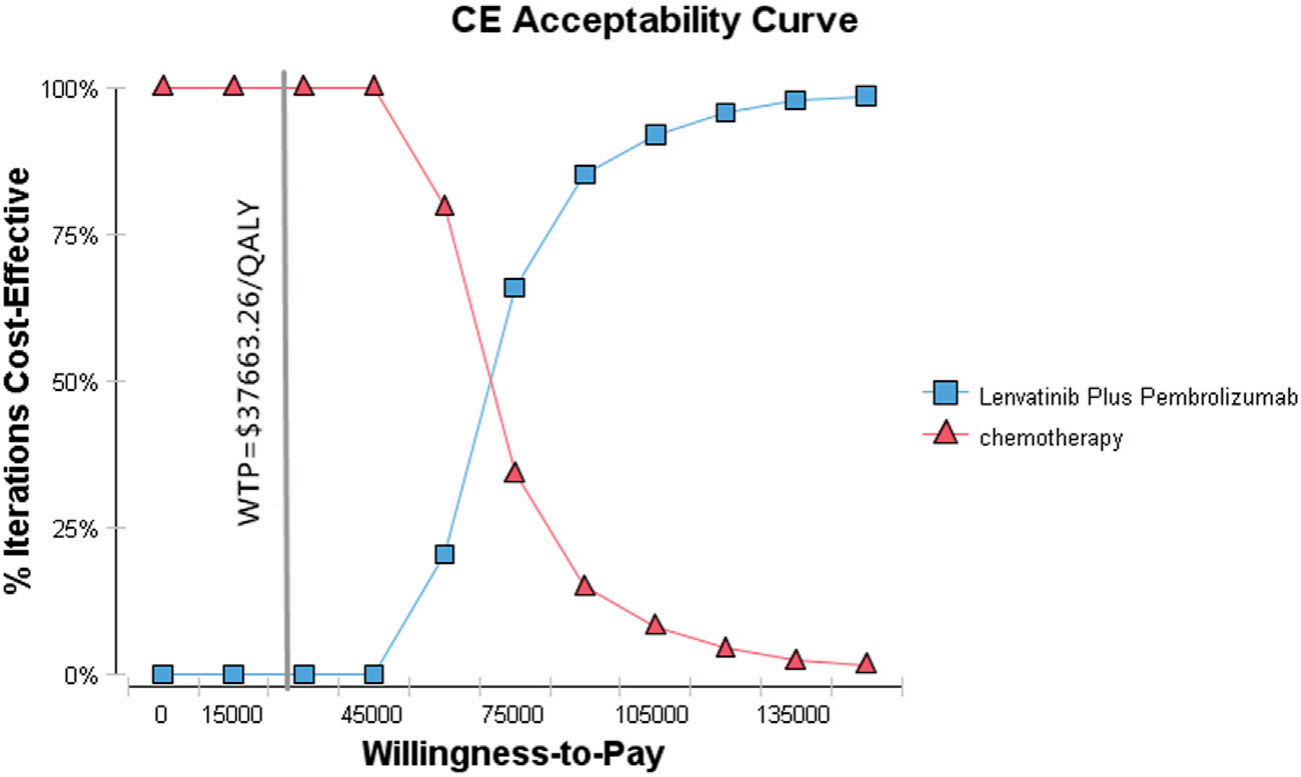
Cost-effectiveness acceptability curve. CE, cost-effectiveness.

**FIGURE 4:**
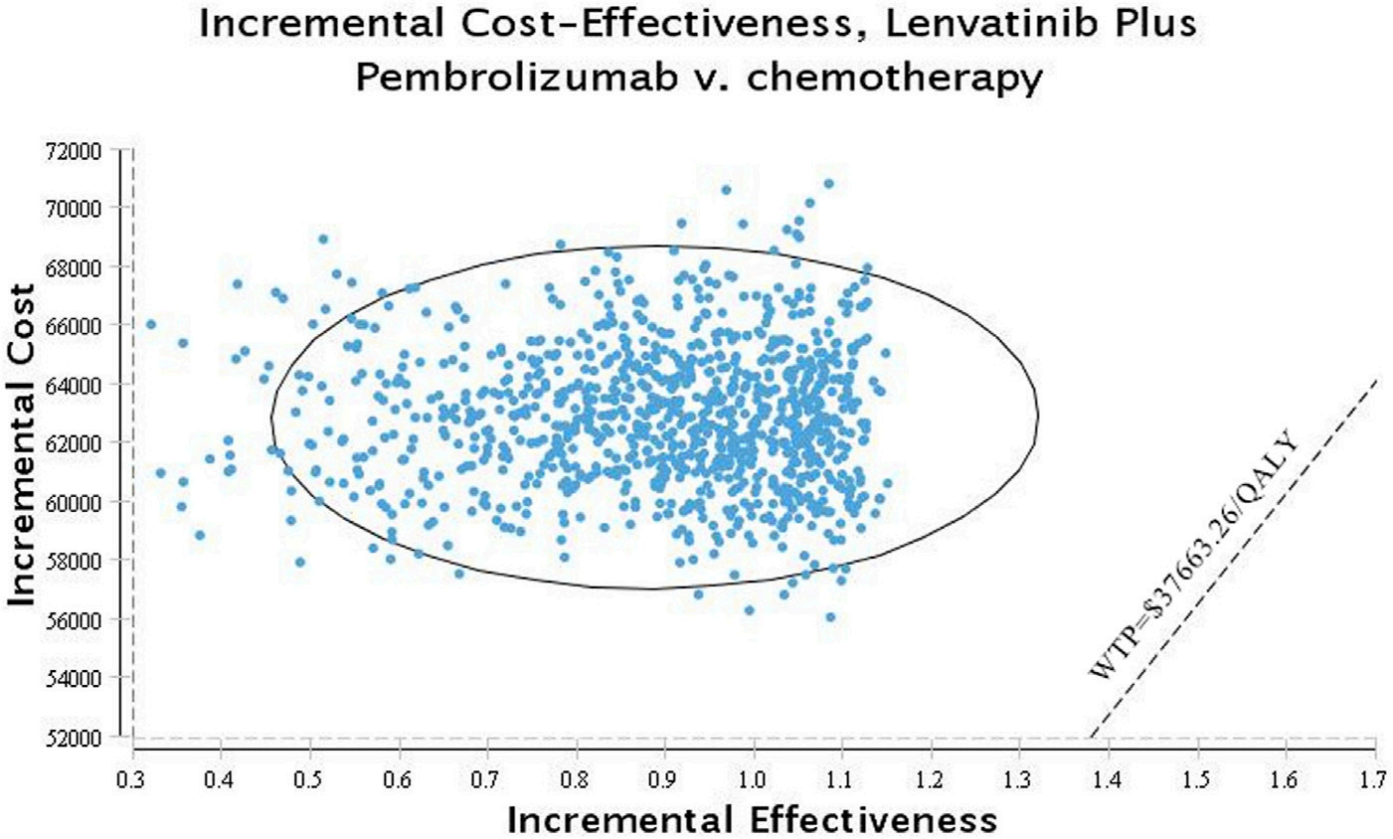
Scatter plot probabilistic scatter plot of the ICER between the lenvatinib plus pembrolizumab group and chemotherapy group.

Figures 3, 4 show the results of the probabilistic sensitivity analysis as a cost-effectiveness acceptability curve and a probabilistic scatter plot. The cost-effectiveness acceptability curves display the effectiveness of probabilistic sensitivity analysis, which evaluates the probability of different treatments being evaluated as optimal strategies at various WTP levels. The probabilistic scatter reflects the Monte Carlo simulation outcome, while the elipse represents the 95% confidence interval. The diagonal line represents the WTP value, and the dot below that indicates that the test group has a cost effect as compared to the control group.

## Discussion

Despite advances in multidisciplinary treatment of EC, treatment options for advanced EC are limited and have poor prognosis (Monk et al., 2022). The study 309–KEYNOTE-775 compared lenvatinib plus pembrolizumab to chemotherapy in patients with advanced endometrial cancer who had progressed or recurred after receiving at least one platinum-based chemotherapy regimen. Both progression-free survival and overall survival were significantly longer in the lenvatinib plus pembrolizumab (LP) group than in the chemotherapy group. This finding addresses the need for effective therapy in these patient populations. However, one of the most serious issues that doctors confront is that new therapy alternatives are typically associated with greater prices than previously used treatments.

New therapeutic interventions, such as immunotherapy and molecularly targeted drugs, develop new ways to treat and improve the survival and quality of life of patients (Cheng et al., 2020). However, these new treatments are expensive, contributing to China’s unsustainable rise in healthcare costs (Xiong et al., 2021). Our study revealed that LP was more expensive ($93,496.69 vs. $30,578.04) and produced more health outcomes (3.13 vs. 2.24 QALYs), yielding an ICER of $70,962.09/QALY, which was significantly more than the WTP threshold ($37,663.26/QALY). Both one-way and probabilistic sensitivity analyses indicated that this result was robust to model parameters or assumptions. The results of this analysis indicated that LP migtht not a cost-effective treatment option vs. chemotherapy for patients with previously treated mismatch repair-proficient advanced endometrial cancer in China.

China has launched comprehensive initiatives to coordinate drug procurement in order to decrease drug costs. The cost-effectiveness of PD-1 inhibitors such as pembrolizumab will be improved with the price adjustment. In the one-way sensitivity analysis, the cost of pembrolizumab had the greatest impact on the ICER. When the price of pembrolizumab in China was reduced from $25.98/mg to $4.936/mg, the analysis of the results showed that the ICER ($37,583.12/QALY) was close to the threshold of WTP ($37,663.26/QALY), suggesting LP might be a cost-effective treatment option when compared to chemotherapy alone for patients with previously treated mismatch repair-proficient advanced endometrial cancer in China. In addition, we can observe that if adverse side effects are reduced and managed efficiently, QALYs increase and the ICER for LP decreases. Furthermore, greater understanding of biomarkers for LP therapy response may allow this strategy to be used in individuals who will benefit the most, while minimizing the harm experienced by non-responders (Falzone, et al., 2021). With roughly 30.3% of patients with pMMR tumors responding to combined therapy with LP, this strategy might help a substantial number of patients. However, 70% of patients will not respond and may experience serious side effects as a result of this treatment. The discovery of a biomarker able to accurately predict the optimum response might increase the strategy’s cost-effectiveness in gynecological malignancies, including ovarian and endometrial cancers (Scott et al., 2020). We can adapt therapy and minimize side effects in nonresponders if we can better anticipate patients’ responses to this pricey, toxic, but very beneficial regimen. We may be able to more accurately administer this medication, enhancing its cost-effectiveness and reducing needless toxicity through multidisciplinary treatment. The identification of predictive biomarkers, multidisciplinary treatment to reduce and manage adverse side effects efficiently, in conjunction with attempts to substantially reduce medication prices, may increase the cost-effectiveness of LP therapy.

A cost-effectiveness analysis of advanced endometrial cancer based on a Chinese payment perspective has not been found in Pubmed to date. To the best of our knowledge, there have been a number of cost-effective assessments comparing treatment for various types of advanced endometrial cancer from a US healthcare payer perspective (Barrington et al., 2019; Ackroyd et al., 2021). A recent cost-effectiveness analysis of pembrolizumab in advanced recurrent endometrial cancer was based on the KEYNOTE-158 trial in the US (Thurgar et al., 2021). The result of that study revealed an ICER of $1,58,907/QALY for pembrolizumab compared with chemotherapy at the WTP threshold of $10,000/QALY. Their conclusion was that pembrolizumab is not a cost-effective treatment option vs. chemotherapy for women with previously treated deficient mismatch repair (dMMR) or high microsatellite instability (MSI-H) advanced endometrial cancer in the US. Ackroyd et al. (2021) designed a Markov model to determine the cost effectiveness of pembrolizumab plus lenvatinib (PL) compared with carboplatin plus paclitaxel (CT) as first-line systemic therapy for patients with advanced or recurrent endometrial cancer based on KEYNOTE-146 in the US. They found PL improved survival and QALYs vs. CT but was not cost-effective in the US. Our study differs from these previous reports in the following ways: First, the primary population in our study is patients with previously treated mismatch repair proficient advanced endometrial cancer; second, we used a partitioned survival approach to determine the probability of metastasis; and third, our study is a cost-effectiveness analysis studied from a Chinese payment perspective.

Our study complied with the consolidated health economic evaluation reporting standards 2022 (Husereau et al., 2022). The study perspective, time range, hypotheses, and sources of validity evaluation were all well described, as well as the patient characteristics of the base case practical suggestions.

However, there are several limits to our analysis. First, the key clinical data in the study was gathered through clinical trials, which would have led to some bias. We calculated treatment duration in our model by extrapolating from the median number of LP cycles reported in the 309-KEYNOTE-775 study. The median number of cycles may, however, be less than the real number of cycles given that certain patients may have long-lasting responses to LP and may continue therapy for up to 35 months. Utilizing the median number of cycles as opposed to the real number of cycles may exaggerate the LP’s cost-effectiveness since it may underestimate the quantity of therapy required to provide the observed survival advantage. Second, we assumed that patients had an equal chance of receiving doxorubicin or paclitaxel for chemotherapy, which is not the usual situation. The sensitivity analysis revealed that the chemotherapy selection option had no significant positive effect on outcomes. Finally, only grade 3/4 SAEs were considered in the analysis. We hypothesized that grade 1/2 SAEs would not influence the study results’ ultimate conclusion, and sensitivity results demonstrated that the result was not sensitive to SAEs-related characteristics. Despite these limitations, our findings may be helpful to Chinese doctors and policymakers.

We would caution readers not to consider this data as a reason to avoid using LP. We think that cost-effectiveness evaluations in cancer treatment should not be taken as evidence to limit the use of effective therapy, but rather as a tool to guide the development of scientific and reasonable prices for drugs and develop a medical insurance drug catalog.

## Conclusion

Compared with chemotherapy, LP was not considered as cost-effective treatment option for patients with previously treated mismatch repair proficient advanced endometrial cancer in China. However, LP may be a cost-effective treatment option if the price is reduced.

## Data Availability

The original contributions presented in the study are included in the article/Supplementary Material, further inquiries can be directed to the corresponding authors.
