# Supplementary figures and images for: Cost-effectiveness analysis of lenvatinib plus pembrolizumab compared with chemotherapy for patients with previously treated mismatch repair proficient advanced endometrial cancer in China
Source: Front Pharmacol. 2022 Sep 30;13:944931. doi: 10.3389/fphar.2022.944931 (PMC9561308; doi:10.3389/fphar.2022.944931)

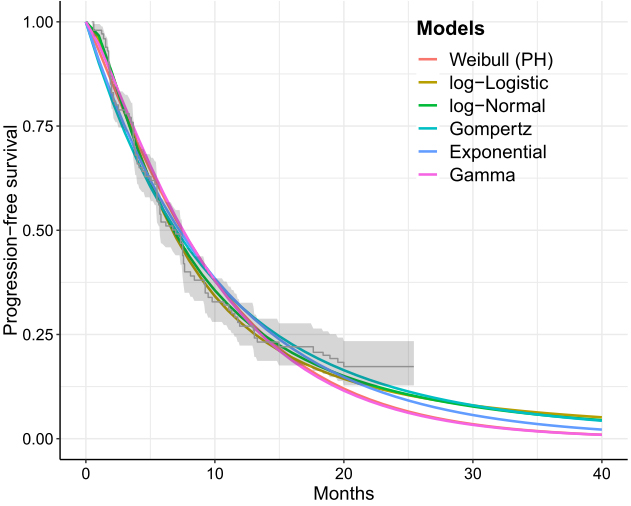

Supplement: Supplementary file 1 [file Image3.JPEG]

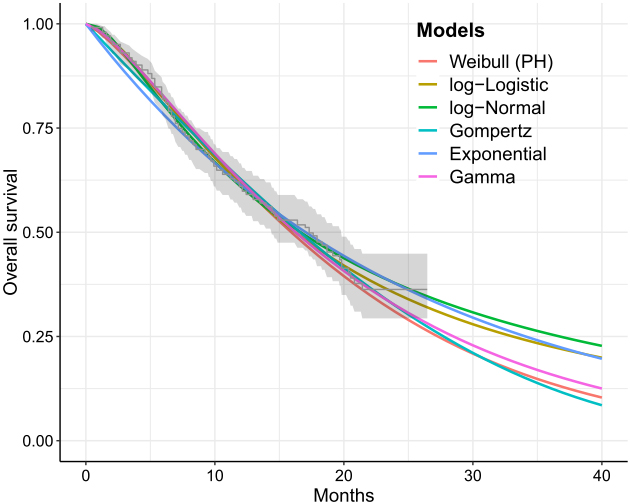

Supplement: Supplementary file 3 [file Image1.JPEG]

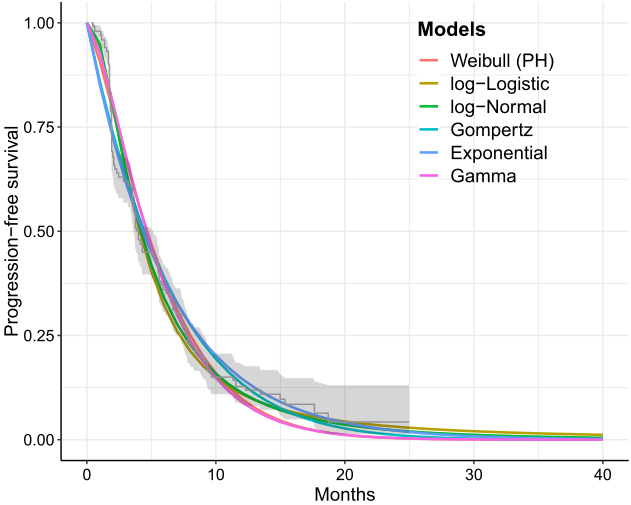

Supplement: Supplementary file 4 [file Image4.JPEG]

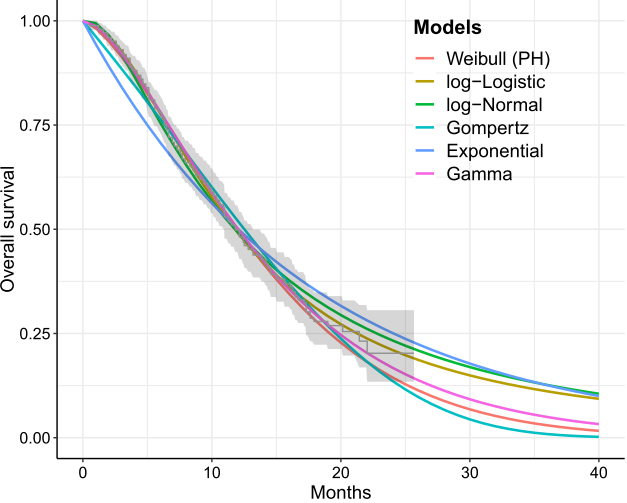

Supplement: Supplementary file 5 [file Image2.JPEG]
