# Supplementary material for: Cost-effectiveness analysis of lenvatinib plus pembrolizumab compared with chemotherapy for patients with previously treated mismatch repair proficient advanced endometrial cancer in China
Source: Front Pharmacol. 2022 Sep 30;13:944931. doi: 10.3389/fphar.2022.944931 (PMC9561308; doi:10.3389/fphar.2022.944931)
Supplement: Supplementary file 2 [file Table1.DOCX]

**Supplementary Table 1. Comparison of survival models**

|  | AIC | | BIC | |
| --- | --- | --- | --- | --- |
|  | LP | Chemotherapy | LP | Chemotherapy |
| OS |  |  |  |  |
| Weibull | 1404.829 | 1556.888 | 1412.522 | 1564.610 |
| **Log-logistic** | **1400.496** | **1553.522** | **1408.189** | **1561.244** |
| Log-normal | 1402.735 | 1561.938 | 1410.428 | 1569.659 |
| Gompertz | 1412.588 | 1572.686 | 1420.281 | 1580.408 |
| Exponential | 1415.079 | 1591.281 | 1418.926 | 1595.142 |
| Gamma | 1402.992 | 1554.296 | 1410.685 | 1562.018 |
| PFS |  |  |  |  |
| Weibull | 1659.797 | 1358.365 | 1667.490 | 1366.086 |
| **Log-logistic** | **1618.791** | **1307.716** | **1626.484** | **1315.438** |
| Log-normal | 1625.013 | 1318.068 | 1632.706 | 1325.790 |
| Gompertz | 1664.009 | 1380.303 | 1671.702 | 1388.025 |
| Exponential | 1663.401 | 1379.517 | 1667.248 | 1383.378 |
| Gamma | 1653.395 | 1344.468 | 1661.088 | 1344.468 |

LP: lenvatinib plus pembrolizumab; AIC: Akaike information criterion; BIC: Bayesian Information Criterion; OS: Overall survival; PFS: Progression-free survival;
